# The Educare intervention: Outcomes at age 3

**DOI:** 10.1016/j.ecresq.2020.05.008

**Published:** 2020

**Authors:** Noreen Yazejian, Donna M. Bryant, Laura J. Kuhn, Margaret Burchinal, Diane Horm, Sydney Hans, Nancy File, Barbara Jackson

**Affiliations:** a University of North Carolina at Chapel Hill, United States; b University of Oklahoma-Tulsa, United States; c University of Chicago, United States; d University of Wisconsin-Milwaukee, United States; e University of Nebraska Medical Center, United States

**Keywords:** Infants and toddlers, Intervention, Achievement gap, Center-Based care

## Abstract

The effectiveness of Educare, a center-based early education program, in improving child outcomes at age 3 was evaluated in a randomized clinical trial. Educare programs serve children from birth to age 5 and are designed to reduce the achievement gap between children from low-income families and their more advantaged peers. This study began following 239 children from low-income families who were randomly assigned as infants (<19 months) to Educare or a business-as-usual control group. At age 3, assessments of the skills of 202 children remaining in the study revealed significant differences in favor of children in the treatment group on auditory language skills, early math skills, and parent-reported problem behaviors. Effect sizes were in the modest range, although not as strong as the previously reported age 2 findings. No effects were found for observations of parent-child interactions, observer-rated child behaviors, or parent-rated social competence. For English-language skills, children who were dual language learners (DLLs) benefitted more from treatment than non-DLLs. Analyses of outcomes by child care type, regardless of treatment group assignment, showed that children in Educare had better language, math, and behavioral scores than children in less formal care, whereas children in other center-based care only had higher language scores than children in less formal care. Differences in outcomes between Educare and other center-based care were not significant.

## Highlights

• A randomized study examined age 3 effects of a high-quality early education program.
• Treatment children had better language, math, and behavior skills than controls.
• There were no treatment effects for parent-child interactions.
• For English-language and math skills, dual language learners benefitted more.
• Regardless of treatment group, children attending centers did better than others.

## 1 Introduction

The achievement gap between children from low-income families and their more affluent peers has been a pernicious, enduring problem in the U.S. education system (Reardon, 2011). The gap has been documented to start early, when children are as young as nine months of age (Halle et al., 2009), and does not change as children progress through primary and secondary schooling (Duncan & Magnuson, 2011). Providing high-quality early care and education (ECE) starting in infancy may give children a boost to ensure they start school on par with their peers (Mathers & Ereky-Stevens, 2018). An experimental study of Educare, a high-quality center-based program designed to address the income gap in early development, reported positive results for children’s language and social-emotional skills and parent-child interactions when children averaged 2 years of age (Yazejian et al., 2017). This paper builds on those initial findings and presents study results when children were age 3 to see whether Educare effects have continued.

Educare is an enhanced Early Head Start (EHS)/Head Start (HS) program designed to provide high-quality birth to age 5 ECE for low-income families. As described in more detail elsewhere (Yazejian et al., 2017; Yazejian, Bryant, & Kennel, 2013), Educare is designed to deliver high-quality classroom and family programming by offering full-day, year-round comprehensive services through blended funding mechanisms. The model is designed to support children’s school readiness by using what is known from the ECE research base in conjunction with HS/EHS performance standards (Office of Human Development Services, 2015).

### 1.1 Need for high-quality early learning environments for disadvantaged children

Demographic statistics and research evidence about children and poverty highlight the need for early intervention programs for children from low-income families starting in infancy. Recent estimates suggest that 5.2 million U.S. infants and toddlers under age 3 live in low-income families (<200% of the federal poverty threshold), representing 45% of all U.S. infants and toddlers (Jiang, Granja, & Koball, 2017). The associations between poverty and negative outcomes for children’s achievement, behavior, and school performance have been well documented (Brooks-Gunn and Duncan, 1997, Reardon, 2011). In addition, poverty is associated with many other factors also shown to be associated with delays in language, cognitive, or social-emotional skills. These include health disparities such as low birthweight (LBW), increased child and parent stress and trauma, lack of stimulating home experiences and responsive interactions, and less access to supports (Aikens & Barbarin, 2008; Pechtel & Pizzagalli, 2011; Yeung, Linver, & Brooks-Gunn, 2002; Yoshikawa, Aber, & Beardslee, 2012).

Several studies have shown that high-quality ECE may buffer children from effects of poverty, particularly when examining language and cognitive outcomes in infancy (Burchinal et al., 2000) and preschool (Dearing, McCartney, & Taylor, 2009; Votruba-Drzal, Coley, Koury, & Miller, 2013). Findings related to social-emotional behaviors are more mixed (McCartney et al., 2010; Votruba-Drzal, Coley, Maldonado-Carreño, Li-Grining, & Chase-Lansdale, 2010). Evidence also suggests that dual language learners (DLLs) may particularly benefit from high-quality center-based care (Bloom and Weiland, 2015, Gormley, 2008, Weiland and Yoshikawa, 2013), although few studies of DLLs have included infants and toddlers (Buysse, Peisner-Feinberg, Paez, Hammer, & Knowles, 2014). A previous longitudinal study of Educare children (infants, toddlers, preschoolers) found associations among length of time in care, age of entry, and language and social-emotional outcomes for all children; these associations were stronger for DLL than for English-only children (Yazejian, Bryant, Freel, & Burchinal, 2015). Some research has suggested that African American children may benefit more from preschool experiences (Bassok, 2010; Huang, Invernizzi, & Drake, 2012), but the research is inconclusive. Ladd (2017) summarized four studies of differential Head Start or PreK effects, finding only one study with larger academic impacts for African-American children.

### 1.2 Historical context

During the 1960s and 1970s, scholars and educators established the theoretical and scientific bases for interventions that had the potential to alter young children’s developmental trajectories (e.g., Bloom, 1964, Bronfenbrenner, 1979, Hunt, 1961). These theoretical bases served as the underpinning for most early programs for infants and toddlers living in poverty (Condry, 1983). Review and meta-analytic articles (Barnett, 1995; Duncan & Magnuson, 2013; Karoly, Kilburn, & Cannon, 2005) have previously summarized the findings on ECE interventions by identifying magnitude of effects seen across areas of child development and family functioning; an extensive review will not be included here. However, three rigorous, random-assignment studies of programs from these decades are worth noting because of their similarity to Educare, that is they spanned the birth-to-5 age range, were targeted to children from families living in poverty, and included some family engagement/home-based components: the Abecedarian study (Campbell & Ramey, 1994), Project CARE (Wasik, Ramey, Bryant, & Sparling, 1990), and the Syracuse Family Development Research Program (Honig & Lally, 1982).

The well-known Abecedarian study examined primarily center-based care that was supplemented with monthly parent meetings; Project CARE compared three groups—center-plus-home visiting, home visiting only, and control; and the Syracuse program involved center- and home-based services from birth for the treatment group and a matched comparison sample that was recruited at 36 months. All three studies reported significant center-care effects on child outcomes at age 3, with effect sizes ranging from 0.9 to 1.1. Additionally, the Abecedarian and CARE studies found center-care effects at age 5 (effect sizes around 0.5). None of the three studies reported differences in parent-child interactions.

Based partly on findings from these studies of infants and toddlers, as well as results showing that effects of HS were modest because children entering at ages 3 and 4 were already evidencing gaps in achievement, the Federal Government scaled two infant and toddler interventions—Comprehensive Child Development Programs (CCDPs) and EHS. The CCDPs primarily implemented a case management approach and built on existing community programs to enhance infant and toddler development. A large, multi-site evaluation of CCDPs found few positive impacts by age 3 for participating families compared with controls; both groups showed some improvement in outcomes (St. Pierre, Layzer, Goodson, & Bernstein, 1997).

EHS began in 1995 for pregnant women and children birth to age 3 with the goals of enhancing both child development and family functioning. EHS can be delivered through home-based, center-based, or combination options, depending on community assessments of needs. In a multi-site randomized study, EHS was found to have modest positive effects at ages 2 and 3 on language and social-emotional development and some parenting measures, with effect sizes of .10–.20 (Love et al., 2005). Although positive, these results were smaller and relatively short-lived compared to the model early intervention programs described above. Descriptive data on EHS classroom quality has shown that children in center-based programs attended classrooms that were in the mid-range of quality, and that more than 40% of children were in classrooms that scored in the low range in support for learning (ACF, 2015). Analyses of Early Childhood Longitudinal Study-Birth Cohort (ECLS-B) data suggest that infant classrooms may need to meet minimum quality thresholds in order for children to show significant gains in cognitive development (Setodji, Le, & Schaack, 2013).

The studies of model early intervention programs like Abecedarian were conducted in an era when the availability and affordability of infant and toddler center-based child care was limited, especially for families of low income, thus children in the control groups were less likely to be enrolled in ECE programs than children of today. More recently, studies have documented “fade-out” or “catch-up” effects, in which treatment and control groups converge (Lipsey, Farran, & Durkin, 2018; Magnuson, Ruhm, & Waldfogel, 2007; U.S. Department of Health & Human Services, 2010). These studies have typically found convergence among groups after preschool attendance, during early school grades. Hypotheses explaining the convergence have been posited but not thoroughly explored. Some argue that effects fade because of the poor quality of the subsequent environments (e.g., primary school classrooms) children experience (Ansari & Pianta, 2018; Brooks-Gunn, 2003; Curenton, Dong, & Shen, 2015; Currie & Thomas, 2000; Lee & Loeb, 1995). Other researchers suggest that preschool attendees are less likely to qualify for additional instructional services in school because of their improved performance, and conversely that the poorer-performing students who may not have attended preschool may get increased attention and instructional services that allow them to catch up to their initially higher performing peers who attended preschool (Cooper, Allen, Patall, & Dent, 2010; Magnuson et al., 2007). Both explanations argue for documenting all of the child care and school experiences of children in treatment and control groups to help understand the counterfactual and explain whether observed effects persist or converge over time.

### 1.3 Previous research on the Educare intervention

Although it is a HS/EHS program, Educare can be described as a hybrid of the national public program and a model demonstration project. All Educare schools meet HS Program Performance Standards, and as Educare schools, they must meet additional higher standards. EHS classroom teacher:child ratios, for example, must be 1:4 or better, whereas Educare’s standard is 3:8. EHS teachers are required to have at least an Associate’s degree, whereas Educare teachers are required to have a B.A. EHS/HS programs are required to gather and report some data on children and family demographics and on service delivery, whereas Educare schools are required to develop a partnership with a local university or similar agency to utilize a researcher to collect data on children, families, staff, and program services and share these data regularly with leadership and staff for program improvement.

Educare’s framework includes four practices that have shown associations with improved programming and children’s outcomes: data utilization, coaching and ongoing professional development, high-quality teaching and interactions, and strong school-family partnerships. These quality practices are based on the literature on effective features of ECE. For example, robust models of professional development and coaching (Weiland & Yoshikawa, 2013) and regular monitoring of child progress (Fantuzzo, Gadsden, & McDermott, 2011) have been evaluated in pre-K and HS programs and found to relate to positive child outcomes. Although no one study has identified the most effective features of successful ECE programs that consistently improve outcomes for children, the quality features adopted by Educare programs have been implemented in various settings and shown to be related to positive child outcomes.

Research examining the outcomes of children attending Educare schools has been promising. A longitudinal study examined outcomes of more than 5000 children from low-income families enrolled in Educare programs between 2008–2013 (1340 entered before 18 months) and found positive associations between receptive language scores and both earlier age of entry and duration in Educare (Yazejian et al., 2015). Children from English-speaking homes who enrolled early and stayed until kindergarten entry left Educare with language scores near national averages, for example, an effect size of 0.26 on the PPVT for children who entered at age 3 and stayed for 2 years versus 1 year. Stronger associations between language scores and dosage (entry age and duration) were found for DLL children than English-only children, for example a large effect (*d* = 0.53) on the PPVT for children who entered at age 3 and had 2 years of experience versus 1 year and a medium effect (*d* = 0.31) for children who entered at age 2 and had 3 years of experience versus 2 years.

The study reported above controlled for a number of child and family characteristics known to be associated with children’s outcomes to attenuate potential selection bias, but other family selection factors may have determined family enrollment patterns. Because of this weakness in study design, a randomized study of Educare was conducted to test the model’s effectiveness. From 2010−12, 239 children (<19 months) from low-income families were assigned to either Educare or a business-as-usual control group. Assessments of 206 children conducted one year after randomization when children averaged 2 years of age, revealed differences favoring treatment-group children on language skills, parent-reported problem behaviors, and positive parent-child interactions. Effect sizes were modest to medium, ranging from −0.28 to .56 (Yazejian et al., 2017). The current study extends these previous findings by examining outcomes at age 3 of 202 children. In addition, given previous findings related to DLL status and race, the study explores potential child characteristics as moderators of Educare effects. The study also looks at whether intervention and treatment groups may be converging over time, and examines Educare’s association with child outcomes relative to other types of ECE settings as an exploration of the counterfactual.

### 1.4 Research questions

This paper answers the following four research questions related to child and parent outcomes: (1) Did Educare and control groups differ at age 3? (2) Do race or dual language status moderate the effect of Educare on child and parent outcomes? (3) Were the changes in scores between the age 2 and age 3 assessments different between treatment and control groups? And (4) What associations did Educare have with age 3 outcomes and change scores between age 2 and 3 relative to other center-based care or little/no childcare? We hypothesized that Educare children would have higher language, math, executive functioning, and social-emotional skills than children in the control group at age 3, and that effects would be stronger for DLL children and African American children based on previous research. The analyses examining convergence and associations between types of care and outcomes were exploratory, although we did hypothesize that children in Educare would have better outcomes than children in the little/no care group.

## 2 Method

The study was designed by program leaders from the Educare schools, their university-affiliated researchers, and a university-based study coordinating team. Bi-weekly telephone calls, annual meetings, and several webinars were held by the study coordinating team to manage activities and train data collectors. Each program’s Policy Council and each university’s Institutional Review Board reviewed and approved all procedures; regional and national Head Start offices were briefed on the study.

### 2.1 The Educare intervention

The Educare intervention and the research base supporting its practices have been detailed elsewhere (Guss, Norris, Horm, Monroe, & Wolfe, 2013; Yazejian et al., 2015, Yazejian et al., 2013) and will be briefly described here. Currently 21 U.S. cities are home to 24 year-round Educare programs, each housed in a center that includes infant, toddler, and preschool classrooms. Schools are open 8−10 hours/day, and children attend at least 6 h/day. Babies as young as 6 weeks can enroll, and the goal is to serve children through preschool. Transportation is not provided.

Because program improvement through data use is a core feature of Educare, all sites are part of an implementation study. Parent, child, classroom, and program information is collected by a local evaluator who shares data locally for program improvement and with a national data center. At least three times a year, teachers use a curriculum-based child monitoring tool to help them individualize teaching and interactions. They also share results with parents at least twice a year. For program improvement, local researchers share the results of their annual classroom observations and standardized assessments with staff (Guss et al., 2013).

Educare schools use a master teacher model wherein teachers are supervised by master teachers who each serve about four classrooms (and do not have their own classroom). A bachelor’s degree is required for both roles and many teachers and master teachers also have an M.A. degree. Master teachers are responsible for teachers’ ongoing professional development and coaching of research-supported best practices. Educare programs hire staff who speak children’s home language, which is Spanish for about 30% of children across all schools. However, instruction is mostly in English or a combination of English and Spanish.

Strong family support is another key intervention feature. Family support specialists and teachers conduct at least two home visits and two parent conferences a year, and the programs offer many family engagement activities, including meetings, classes, and social events. The goals are to encourage positive parent-child relationships, help parents promote their children’s development, and support family well-being. Local Policy Councils that include parents and community members meet monthly to further engage some parents in program planning.

### 2.2 Study recruitment and participants

This section describes the schools, teachers, parents and children who participated in the study, the recruitment process, and subject attrition.

#### 2.2.1 Sites

Five Educare schools in Chicago, Milwaukee, Omaha (2 schools), and Tulsa participated in the study. They were selected because their programs were well-established and their staff were willing to enroll families selected from a waitlist in a random fashion. Beginning in 2010, families with infants younger than 19 months of age who wanted to enroll in Educare were recruited and randomly assigned to either the Educare or control group at all five sites; two schools recruited study children over a second school year. Three programs served predominantly African American families and two served predominantly Spanish-speaking Hispanic families. The proportion of the total sample each school contributed to this study ranged from 10% to 31.4%.

#### 2.2.2 Classrooms and teachers

At age 3, treatment group children who still attended Educare were enrolled in 31 classrooms across the five sites (site range: 3–12 classrooms) that were similar in terms of structural and process quality characteristics. Some children were still enrolled in an EHS class and others had moved into a HS classroom. The average number of children in the EHS classes was 8.5 and the mean adult:child ratio was 1:2.7. The average number of children in the HS classes was 18.4 and the mean adult:child ratio was 1:6.4. Site-level attendance rates of study children ranged from 76 to 91% with a mean of 85%.

The quality of these classrooms was in the high range according to scores on the age-appropriate Environment Rating Scale (ITERS-R, Harms, Clifford, & Cryer, 1998 and ECERS-R, Harms, Cryer, & Clifford, 2003) and the age-appropriate Classroom Assessment Scoring System (La Paro, Hamre, & Pianta, 2012; Pianta, La Paro, & Hamre, 2008). Means for these measures were: ITERS-R total, 6.2 (*SD* = 0.4); ECERS-R total, 6.3 (*SD* = 0.5); Toddler CLASS Emotional Support, 6.5 (*SD* = 0.3); Toddler CLASS Engaged Support for Learning, 4.9 (*SD* = 0.8); and the Pre-K CLASS scales of Emotional Support, 6.6 (*SD* = 0.3), Classroom Organization, 6.0 (*SD* = 0.5), and Instructional Support, 4.1 (*SD* = 0.7). Continuity of care is highly emphasized in Educare, but its implementation varies across sites (Horm et al., 2017). With “continuity” defined as having the same lead teacher during the years until age 3, 45% of children had continuity (range across schools was 18–94%).

Education and experience data were available for 37 lead and 36 assistant teachers; a few classrooms had co-lead teachers. All lead teachers had a BA degree and 32% also had an MA. Among assistants, 44% had a BA and 50% had an AA. Mean years of ECE experience was 12.9 (*SD* = 9.8) for teachers and 11.3 (*SD* = 7.4) for assistants. Mean years of Educare experience was 4.2 (*SD* = 3.2) for teachers and 3.8 (*SD* = 3.1) for assistants. Seven (18.9%) teachers and 10 (27.8%) assistants were in their 1^st^ year at Educare, and 76.5% of the classrooms with a Spanish-speaking DLL child had a lead or assistant who spoke Spanish. All master teachers for these classes had a BA, 88% had an MA, and they served an average of 3.9 classrooms.

#### 2.2.3 Children and families

Recruitment and random assignment procedures were detailed in the age 2 paper (Yazejian et al., 2017) and will be summarized here. Following their standard procedures, family support staff met with parents interested in Educare to ensure that they met the EHS poverty criterion (below federal poverty limit based on family size), had a child who met study criteria (<19 months of age and not an enrolled child’s sibling, a staff child, or a foster child), and agreed to be randomized to treatment or control groups. Family support recruiters reported that 90–95% of eligible families agreed to meet with the research assistant (RA) for study enrollment. The executive director at each school had discretion to enroll two infants who would not be included in this study. Across the five schools, only two exception slots were used.

EHS requires programs to prioritize families by locally determined risk indicators, and the family support recruiters had collected these data. Beginning in the fall of 2010, when openings occurred in infant or toddler classes, the highest risk families recruited by family support staff were contacted by a trained RA to explain the study. If consent was obtained, the RA collected baseline data and, at the end of the visit, called the coordinating research site to find out the child’s randomly assigned group. The study statistician had used blocked randomization to create a predetermined order of treatment or control. Only the central research site knew the next-up assignment. The site RAs used scripts to inform parents of their group assignment and gave lists of other ECE resources to control group parents, encouraging them to remain in the study.

**Fig. 1:**
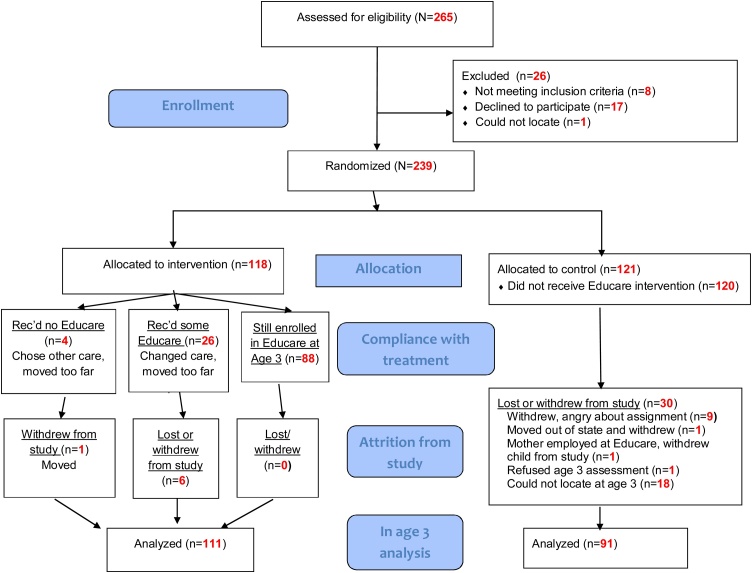
Consort diagram.

Of the 265 children and families referred by family support staff and further assessed for eligibility by the RAs, 8 were found to be ineligible for Educare (e.g., no means of transportation, family income had increased); 17 retracted their initial agreement to be in the study; and one could not be found (see Fig. 1). Ultimately, 239 infants and toddlers (<19 mos.) were randomly assigned to Educare (*n* = 118) or the control group (*n* = 121). Child and family characteristics at enrollment are presented in Table 1. About half were girls; somewhat more than half were African American; over 1/3 identified as Hispanic; and the mothers had, on average, an 11th grade education. Forty-five percent screened positive on a short depression screener (RAND Health, 1998), and about 22% were working full-time. Most children had previously received non-parental child care, averaging about 7 months across all children; about 25% had already been in center-based care for some period of time before entering the study. T-tests and chi-squares conducted on these variables found that treatment and control groups significantly differed on only one baseline characteristic—the parent-reported birthweight tended to be higher for children in the Educare group than in the control group (*p* = 0.04).

**Table 1:** Baseline characteristics of recruited sample (*N* = 239).

	Study group							
	Control			Treatment				
	N	Prop./Mean	SD	N	Prop./Mean	SD	*p*	*ES*
*Child characteristics*								
Age at entry (years)	121	0.78	0.40	118	0.78	0.44	.95	0.00
Gender (male)	121	0.45		118	0.53		.18	0.18
Ethnicity (Hispanic)	119	0.32		118	0.44		.06	0.27
Race: Black	112	0.57		114	0.54		.68	0.05
Race: White	112	0.40		114	0.41		.88	0.02
Birth weight (pounds)	113	7.10	1.25	110	7.38	0.99	**.04**	**0.25**
*Family characteristics*								
Only English spoken in household	121	0.64		118	0.63		.77	0.06
Mother was interviewed	121	0.99		118	0.97		.90	0.18
Number in household	121	4.40	1.76	118	4.43	1.52	.90	0.02
Parent education (years)	121	11.08	2.54	118	11.39	2.44	.34	0.12
Mother age (years)	121	25.55	6.85	118	25.15	6.32	.63	0.06
Teen mother (< 19y when child born)	121	0.15		118	0.17		.66	0.05
Mother depressive symptoms	121	0.48		118	0.40		.21	0.16
Mother works full time (35+ hr/wk)	121	0.18		118	0.27		.10	0.21
*Previous child care experiences*								
Had some non-parental child care	88	0.78		93	0.79		.61	0.03
Center-based care	88	0.27		93	0.23		.47	0.01
Informal care	88	0.90		93	0.88		.91	0.01
Mean hours/week	88	29.90	21.28	93	24.79	21.52	.11	0.20
Number of months	88	7.46	5.03	93	6.96	4.89	.50	0.10
*Baseline PLS-4*^a^								
English Auditory Comprehension	82	98.11	14.27	84	98.68	12.45	.78	0.04
Spanish Auditory Comprehension	39	94.08	15.39	34	88.47	13.64	.11	0.39

*Note*: At baseline, PLS-4 English was not administered to children who spoke only Spanish, and PLS-4 Spanish was not administered to children who spoke only English.

a PLS-4 = Preschool Language Scale, 4th edition.

#### 2.2.4 Attrition

Fig. 1 documents attrition and treatment compliance between enrollment and the age 3 assessment when 202 children (85% of the original sample) participated in the study. Within the treatment group, four families never attended Educare, although three of those remained in the study; one family moved away and withdrew from the study. Twenty-six treatment children were not enrolled in Educare at age 3 but had attended for a period ranging from 1 to 32 months. Twenty of these 26 families remained in the study at age 3; six families had withdrawn, either because they moved very far away and withdrew from the study (*n* = 2), changed child care and withdrew from the study (*n* = 1), or could not be located at age 3 (*n* = 3). Among control families, nine withdrew from the study almost immediately due to disappointment at not being assigned to Educare, one moved out of state and withdrew, one refused the age 3 assessment, and 18 could not be located. One control group mother became employed at Educare and was allowed to enroll her child, but then withdrew from the study. One control child was allowed to enter Educare 2 months before his age 3 assessment. Thus, 91 of 121 (75%) control group children and 111 of 118 (94%) treatment group children participated in the study at age 3.

### 2.3 Data collection procedures

At the pre-randomization visit, trained RAs conducted parent interviews to collect family demographics and baseline assessments of children’s auditory language skills. Based on parent request or support staff recommendation, about 30% of these interviews and assessments were conducted in Spanish. Parents received $20 for this short (30-min.) baseline session. Approximately 3 and 6 months later, researchers made brief (15-min.) calls to parents to maintain contact and gather information on any new child care situations. Parents were sent $10 for these interviews (Yazejian et al., 2017). About one year after randomization, when children averaged 2 years of age (range 13–36 months), parents were interviewed about family characteristics and rated their children’s social-emotional development; children were assessed with measures of expressive and auditory language development; and a parent-child play interaction was recorded.

The current paper uses data gathered at the age 3 visit (*M* = 37.23 months, *SD* = 1.99, range = 36–50 months). Some families were hard to locate or schedule, resulting in assessments of 11 children who were older than 3.5 years. Four treatment and 5 control children were between 3.5–3.75 years old and 2 controls were older than 3.75 years, one of whom was just past the 4th birthday. At the age 3 visit, 38% of the children were DLLs and were assessed in both English and Spanish on language measures; 62% of children lived in English-only homes and were assessed only in English. The age 3 session lasted about 2 h and parents received $100 for the visit. To keep assessors blind to children’s group assignment, sessions were conducted at neutral sites, such as university offices and community agencies.

### 2.4 Measures

The assessment battery administered at age 3 included parent ratings and computer-assisted interviews, observations of parent-child interactions, and direct child assessments. The five domains of outcome measures are described below, representing: (a) language development, (b) math skills, (c) social-emotional development, (d) executive function (EF), and (e) parent-child interactions. We also conducted parent interviews to gather information about background characteristics, child care experiences, and family activities.

#### 2.4.1 Language development

The auditory comprehension subscale of the Preschool Language Scale, Fourth Edition (PLS-4; Zimmerman, Steiner, & Pond, 2002) was used to individually assess language skills at age 3. Both the English and Spanish versions were given to children with home exposure to Spanish. This assessment involves interactive tasks that assess preverbal behaviors, linguistic skills (semantics, morphology, syntax), integrative language skills, and pre-literacy skills. In the current study sample, reliability estimates (Cronbach’s alpha) at age 3 were α = 0.88 for the English auditory scale and α = 0.85 for the Spanish auditory scale. The standard scores were used as the outcome measures.

#### 2.4.2 Math skills

Children’s numeracy skills were measured with the applied problems subtest of the Woodcock-Johnson III Tests of Achievement (WJ-III; Woodcock, McGrew, & Mather, 2001) or for Spanish-speaking children, with the parallel Batería III Woodcock-Muñoz (Woodcock, Muñoz-Sandoval, McGrew, & Mather, 2004). This subtest requires children to analyze and solve math problems and has a median reliability of 0.93 (Schrank et al., 2005). In the current study sample, the reliability estimate at age 3 was α = 0.76 for the English WJ-III AP subtest and α = 0.66 for the parallel Batería subtest. The standard score was used as the outcome measure.

#### 2.4.3 Social-emotional development

Parents rated children’s social skills at age 3 using the Brief Infant Toddler Social Emotional Assessment (BITSEA; Briggs-Gowan & Carter, 2006), a 42-item standardized, norm-referenced screener for children aged 1–3. The BITSEA problem scale (31 items) assesses social-emotional and behavioral problems such as aggression, defiance, and anxiety; higher scores indicate more problems. The competence scale (11 items) assesses prosocial behaviors and compliance; lower scores indicate less competence. The BITSEA authors report excellent test-retest reliability (*r* = 0.79–0.92), good inter-rater reliability (*r* = 0.55–0.78), and adequate internal consistency (Cronbach’s α = 0.79 for the problem scale and α = 0.65 for the competence scale). In our sample, α = 0.84 for the problem scale and α = 0.67 for the competence scale. The BITSEA’s validity was shown by predicting social-emotional behaviors in primary school (Briggs-Gowan & Carter, 2008).

#### 2.4.4 Executive function

The Executive Functions (EF) Touch battery consists of seven computerized tasks that are appropriate for 3- to 5-year-olds and administered via a touch-screen monitor (Willoughby & Blair, 2016). The four tasks used in the current study were Working Memory Span, Silly Sounds Stroop, Spatial Conflict Arrows, and Something’s the Same. The Working Memory Span task requires children to name and hold in memory two pieces of information simultaneously (i.e., the name of a color and an animal) and to activate one while ignoring interference from the other. The Silly Sounds Stroop task presents sounds of cats and dogs and asks children to touch the picture of the animal opposite to that of the sound (i.e., to touch the dog when “meow” is heard). In the Spatial Conflict Arrows task children touch the button corresponding to the direction of a pointing arrow while inhibiting arrow’s spatial location. The Something’s the Same task asks children to select pictures on one dimension (e.g., color) and then shift attention to select a picture on a new dimension (e.g., size). The EF Touch authors have determined that children’s task performance is best represented as a composite mean score (Willoughby, Blair, & Family Life Project Investigators, 2016).

#### 2.4.5 Parent-child interactions

The Two Bags Task used in ECLS-B (Andreassen & Fletcher, 2007) was used to code parent-child interactions. The procedure results in variables reflecting parent and child behaviors and relationship quality, such as responsiveness, hostility, and positive affect. Parents and children were presented with two bags of activities, the first containing a book and the second, a set of toys (cash register and groceries at age 3). The RA asked them to play for 10 min with both activities, opening the book bag first. The sessions were video recorded; recordings were uploaded to the central research site and coded by trained observers who were blind to participants’ study group assignment. A primary coder from the ECLS-B study trained the coders who were then required to score within 1 point of the gold standard on all scales on 4 of 5 test videos before being certified. One coder was English-Spanish bilingual.

Video recordings were coded for 8 dimensions: parent emotional supportiveness, parent stimulation of cognitive development, parent intrusiveness, parent negative regard, parent detachment, child engagement of parent, child quality of play, and child negativity toward parent. These dimensions were analyzed as two parent factors and one child factor based on previous work (Yazejian et al., 2017). Parent Positive was comprised of parent emotional supportiveness, stimulation of cognitive development, and detachment (reversed), Parent Negative was comprised of parent intrusiveness and negative regard and Child Positive was comprised of the three child rating dimensions with child negativity toward parent reversed.

Approximately 18% of the age 3 video recordings were coded independently by two coders. Inter-rater reliability for the factors was examined with intra-class correlation coefficients (ICC) to describe linear associations and with weighted kappas (*k*) to describe levels of agreement taking the underlying distribution into account. Results suggested good reliability: Parent Positive (ICC = 0.84), Parent Negative (ICC = 0.88) and Child Positive (ICC = 0.87).

#### 2.4.6 Parent interview

The computer-assisted parent interviews at age 3 obtained updated information on family and child demographics and family activities. Parent interviews also included questions about the types and hours of non-parental child care since the last data collection time-point. Most children had experienced some type of non-parental child care before study entry, 78% of the control group and 79% of the treatment group. Among the control children, 27% had been in center care and 90% in informal care (family child care home or family/friend/neighbor care). Among the treatment group children, 23% had been in center care and 88% in informal care before study entry. In both groups, 16% had experienced multiple concurrent arrangements. Including any type of care, control children averaged 30 h/week in care for about 7 months before they joined the study, and treatment children averaged 25 h/week for 7 months (Yazejian et al., 2017).

### 2.5 Analysis plan

Four sets of analyses addressed the four research questions. To test whether Educare and control groups differed at age 3, both intent-to-treat (ITT) and per protocol (PP) analyses were conducted with two-level hierarchical linear modeling (HLM). The ITT analyses tested whether being assigned to Educare, even if a child never attended Educare, was associated with higher scores at age 3 in the domains of language, math skills, social-emotional development, EF, and parent-child interactions compared to children assigned to the control group. The PP analyses tested whether children who were assigned to and attended Educare through age 3 were different than control group children.

The second research question was addressed with HLM analyses that tested whether the Educare effect was moderated by certain child factors, testing each interaction in separate ITT analyses. Another set of ITT analyses tested the third question, whether attending Educare was related to larger change scores in child outcomes between ages 2 and 3 years compared to children in the control group.

For all the analyses noted above, and for each outcome variable, nested models were fit to account for nesting of children within the five Educare schools. Using Proc Mixed in SAS 9.4, the model specified random intercepts for the Educare schools. The models included treatment as the predictor of interest and several covariates. Birthweight was included as a covariate to account for baseline group differences. Child age at both program entry and assessment; gender; ethnicity (African American/Hispanic); home language; and mother’s age, education, work hours, and depression screen status were also included as covariates to reduce variability. Effect sizes were computed as the difference between the treatment and control group adjusted means as estimated in the HLMs, divided by the control group standard deviation.

The fourth set of analyses disregarded the initial group assignments and compared the outcomes of children by their center-based care experience. For these analyses, children were assigned to an Educare group or other center-based care group if they had attended that program 1,200 h or more as of the age 3 assessment. Considering the distribution of care hours across the sample, no obvious cut-point appeared, so a threshold of 1,200 h was chosen because it approximated one year of attendance (48 weeks × 25 hours/week). To form groups of sufficient size, EHS/HS and other community child care programs were combined into one category—other center-based care. Children who had attended a center-based program for less than 1,200 h by age 3 or who had been in family child care homes for any number of hours or who had only parental care were combined into a third group—little or no center-based child care. Two-level hierarchical linear modeling (HLM) was conducted as above, but substituting center-based care type for treatment group. The first of these looked at the effect of care type on age 3 outcomes and the second looked at age 2 to age 3 change scores.

Multiple imputations were conducted in all analyses to account for the small amount of missing data. Scores from the parent-child interaction were missing from 11%; birthweight was missing from 6%, and all other variables were missing 0-5%. The imputation model included site, baseline family and child characteristics, and child outcomes at baseline and age 3. For each missing value, a set of plausible values was estimated from the imputation model using a Bayesian E-M algorithm with bootstrapping (Schafer, 1997) to produce 40 imputation data sets. Analyses were conducted with each data set, and results were combined taking into account variability within and between data sets.

## 3 Results

### 3.1 Attrition and descriptive statistics

Children entered the study at 9.5 months on average. Table 1 presents child and family demographic and child care data at study enrollment. When children were about 3 years of age, 85% (*n* = 202) of the original sample of 239 children participated in an assessment; 2 had only a parent interview. Across both groups, children who left the study by age 3 were more likely to have lower baseline PLS scores (left = 93.85, remained = 99.28, *p* < .05) and to have mothers who screened positive for depression at baseline (left = 59%, remained = 41%, *p* < .05). Rates of attrition by treatment group assignment differed significantly (χ^2^ = 16.24, *p* < .001), with 30 out of 121 children in the control condition (25%) versus 7 out of 118 children in the treatment group (6%) having left the study by age 3.

Table 2 includes the child and family demographic characteristics of the age 3 sample. In addition to birthweight, at this point in time the two groups differed significantly on ethnicity with significantly more children identified as Hispanic in the treatment group than in the control group. There were also large site differences in the proportion of DLL children, so site and DLL status, as well as birthweight, are included in all analyses. Means and standard deviations of child outcomes and parent-child interaction ratings for treatment and control groups at age 3 are also presented in Table 2. Correlations among outcome measures are presented in Table 3.

**Table 2:** Descriptive statistics of analytic sample at age 3 (*N* = 202).

	Study group							
	Control				Treatment			
	N	Prop./Mean	SD	N	Prop./Mean	SD	*p*	*ES*
*Child characteristics @ baseline*								
Age at entry (years)	91	0.80	0.39	111	0.77	0.44	.53	0.07
Gender (male)	91	0.44		111	0.53		.20	0.18
Ethnicity (Hispanic)	90	0.28		111	0.43		**.04**	**0.33**
Race: Black	86	0.59		107	0.55		.56	0.08
Race: White	86	0.38		107	0.40		.80	0.04
Birth weight (pounds)	86	7.07	1.30	104	7.37	1.01	**.05**	**0.26**
*Family characteristics @ baseline*								
Only English spoken in household	91	0.67		111	0.62		.47	0.13
Mother was interviewed	91	0.99		111	0.96		.25	0.17
Number in household	91	4.44	1.69	111	4.44	1.46	.99	0.00
Parent education (years)	91	11.21	2.42	111	11.35	2.49	.68	0.06
Mother age (years)	91	25.91	7.44	111	25.33	6.39	.55	0.08
Teen mother (< 19y when child born)	91	0.14		111	0.16		.70	0.05
Mother depressive symptoms	91	0.42		111	0.41		.86	0.03
Mother works full time (35+ hr/wk)	91	0.18		111	0.26		.15	0.21
*PLS-*4^a^*@ baseline*								
English Auditory Comprehension	82	98.11	14.27	84	98.68	12.45	.78	0.04
Spanish Auditory Comprehension	39	94.08	15.39	34	88.47	13.64	.11	0.39
*Age 3 child outcomes*								
Child age at assessment	91	3.16	0.2	111	3.14	0.15	.47	0.11
PLS-4 English Auditory Comp.	90	89.74	19.31	110	94.07	16.17	.09	0.24
PLS-4 Spanish Auditory Comp.	32	85.75	14.30	44	88.55	15.13	.42	0.19
WJ-3^b^ Applied Problems stnd. score	90	85.41	13.64	110	88.30	14.06	.16	0.20
Executive function battery score	85	.38	.14	107	.36	.12	.43	0.15
BITSEA^c^ Problems	91	12.18	7.04	111	10.09	6.54	**.03**	**0.31**
BITSEA Competence	91	18.38	2.80	111	18.64	2.22	.48	0.10
*Age 3 parent-child interaction*								
Parent Positive factor	82	4.80	.95	98	5.00	.72	.12	0.24
Parent Negative factor	82	1.82	.73	98	1.80	.83	.85	0.03
Child Positive factor	82	4.81	.80	98	4.95	.82	.24	0.17

a PLS-4 = Preschool Language Scale, 4th edition.

b WJ = Woodcock-Johnson Tests of Achievement-3.

c BITSEA = Brief Infant Toddler Social Emotional Assessment.

**Table 3:** Correlations among age 3 outcomes.

Measure	1	2	3	4	5	6	7	8	9
1. PLS-4^a^ Eng Auditory	--	0.36**	0.60***	0.38***	−0.25***	0.21**	0.14	0.08	0.16*
2. PLS-4 Span Auditory			0.35***	0.18	−0.13	0.25*	0.10	−0.34**	0.24*
3. WJ-3^b^ Appl Problems				0.33***	−0.10	0.12	0.18**	−0.10	0.19*
4. Exec Function Total					−0.08	0.12	−0.02	0.03	0.02
5. BITSEA^c^ Problems						−0.37***	−0.07	0.15*	−0.08
6. BITSEA Competence							0.14	−0.02	0.18**
7. Parent Positive								−0.47***	0.58***
8. Parent Negative									−0.47***
9. Child Positive									--

a PLS-4 = Preschool Language Scale, 4th edition.

b WJ-3 = Woodcock-Johnson-3.

c BITSEA = Brief Infant Toddler Social Emotional Assessment.

* *p* < 0.05.

** *p* < 0.01.

*** *p* < 0.001.

Non-parental ECE experiences, including family engagement activities in center-based care, were examined descriptively (see Table 4). Between birth and the age 3 visit, 96% of the treatment group children attended Educare for at least some time, and 77% were also cared for in informal settings (family child care, relatives, or babysitters). Some left Educare before age 3 to enroll in other child care centers and some attended other programs while enrolled in Educare (e.g., evening care). Among control group children, 8% had only parental care between birth and age 3. Of those who had some non-parental care, 90% had been in informal care for some period of time and half had experienced some center-based care, although only 45% were in center care at the age 3 assessment. Between birth and age 3, children in both groups experienced a number of different care settings for many months and hours per week. On average treatment children had been in 2.7 different care settings (range = 1–6) and control children, 2.9 (range = 1–7).

**Table 4:** Child care experiences of analytic sample (*N* = 202).

	Study group					
	Control (n = 91)			Treatment (n = 111)		
	N	Mean or %	SD	N	Mean or %	SD
*Care experiences between birth & age 3*						
Had some non-parental child care, %	84	92.3		111	100.0	
Educare, %	1	1.0		107	96.4	
EHS/HS or public Pre-k, %	12	14.3		3	2.7	
Other center-based, %	42	50.0		30	27.0	
Informal child care, %	76	90.0		85	76.6	
Total # care settings, mean	84	2.9	1.5	111	2.7	1.3
Months in care, mean	84	31.9	8.8	111	33.1	5.6
Hours/week in care, mean	84	35.1	20.0	111	38.9	14.0
*Primary care type and hours*						
Educare (≥ 1200 h)						
Months, mean	0	0	0	98	26.5	6.0
Hours/week in care, mean	0	0	0	98	34.6	6.1
Other Center-based (≥ 1200 h)						
Months, mean	41	22.1	9.4	8	22.8	12.0
Hours/week in care, mean	41	39.3	14.7	8	33.4	9.0
Informal + Little or no center care (< 1200 h)						
Months, mean	43	31.1	9.5	5	28.6	13.8
Hours/week in care, mean	43	28.0	20.6	5	22.1	16.1
Parental Care Only	7	–	–	0	–	–

### 3.2 Treatment analyses

Four sets of analyses addressing the research questions are described below.

#### 3.2.1 Treatment effects on age 3 outcomes

Results presented in Table 5 indicate treatment differences on three of the nine outcomes analyzed with 2-level HLMs that included random intercepts for sites and age at program entry, age at testing, birthweight, maternal depression, maternal education, maternal work hours, maternal age, child sex, DLL, African-American, and Hispanic as covariates. Compared to control children, Educare children had significantly higher English language skills (PLS-4 Auditory Comprehension, *B* = 4.36, *se* = 2.15*, p* < 0.05, *d* = 0.24), higher math scores (WJ-3 Applied Problems, *B* = 3.93, *se* = 1.79*, p* < 0.05, *d* = 0.28), and fewer parent-reported behavior problems (BITSEA Problems, *B* = −2.39, *se* = 0.94, *p* < 0.05, *d* = −0.35). No treatment effects were found for Spanish language skills, EF, or the maternal-child interaction factor scores.

**Table 5:** Hierarchical linear model results: age 3 intent to treat analyses.

	PLS-4^a^ Eng AC		PLS-4 Span AC		EF total		WJ-3^b^ AP		BITSEA^c^ Problems		BITSEA^c^ Competence		Parent Positive		Parent Negative		Child Positive	
Fixed Effect	B	SE	B	SE	B	SE	B	SE	B	SE	B	SE	B	SE	B	SE	B	SE
Educare	4.36*	2.15	1.28	3.77	−0.03	0.02	3.93*	1.79	−2.39*	0.94	0.35	0.35	0.14	0.14	0.03	0.15	0.09	0.15
Child age at entry	4.33	2.73	0.89	4.56	0.01	0.03	1.54	2.37	−2.12	1.18	−0.28	0.46	−0.19	0.20	0.18	0.17	−0.03	0.19
Age at assessmt	−9.56	6.70	−12.39	11.78	0.04	0.07	−4.98	5.37	2.82	3.09	−0.54	1.13	1.27*	0.55	−0.80	0.52	0.64	0.60
Birth weight	1.25	0.99	−0.36	1.52	0.00	0.01	0.06	0.79	−0.15	0.45	0.03	0.16	−0.06	0.07	0.04	0.06	0.01	0.07
Depression	1.34	2.15	1.97	3.68	0.02	0.02	−1.35	1.83	1.57	1.00	0.01	0.37	−0.06	0.16	0.09	0.14	−0.10	0.15
Mother educ	0.89	0.49	−0.82	0.68	0.01	0.00	0.83*	0.43	−0.12	0.22	0.11	0.08	0.07*	0.03	−0.01	0.03	0.05	0.03
Mother hrs wrk	−0.04	0.06	0.14	0.11	0.00	0.00	−0.11*	0.05	0.00	0.03	0.00	0.01	0.00	0.00	0.00	0.00	0.00	0.00
Mother age	−0.23	0.17	0.19	0.26	0.00	0.00	−0.15	0.14	−0.14	0.08	0.03	0.03	0.01	0.01	0.00	0.01	−0.01	0.01
Male	−4.98*	2.14	0.95	3.61	0.01	0.02	−1.44	1.82	1.85*	0.96	−1.05	0.36	−0.01	0.14	0.10	0.14	−0.17	0.14
DLL	−14.46**	4.59	–	–	−0.04	0.05	−11.58**	4.07	0.64	2.08	−0.66**	0.76	−0.08	0.35	−0.08	0.35	−0.36	0.34
African American	2.32	5.28	−20.38	31.27	−0.01	0.04	−7.14	4.78	0.05	2.18	0.06	0.79	−0.26	0.36	0.68*	0.34	−0.38	0.38
Hispanic	6.10	3.59	9.06	4.84	0.03	0.03	−0.63	2.98	1.20	1.56	0.04	0.56	−0.01	0.24	0.23	0.22	0.13	0.22
*Adjusted Means*																		
Control	90.13	2.32	87.28	3.29	0.39	0.02	84.72	2.40	12.08	1.00	18.38	0.30	4.88	0.12	1.72	0.12	4.93	0.14
Educare	94.49	2.23	88.56	2.91	0.36	0.01	88.65	2.31	9.69	0.98	18.73	0.29	5.02	0.11	1.75	0.09	5.01	0.11

a PLS-4 = Preschool Language Scale, 4th edition.

b WJ-3 = Woodcock-Johnson-3.

c BITSEA = Brief Infant Toddler Social Emotional Assessment.

* *p* < 0.05.

** *p* < 0.01.

The per protocol analysis that compared the group of children who were assigned to and attended Educare through age 3 (*n* = 88) with the control group children (*n* = 91) yielded similar results as the ITT analyses, as shown in Table 6. The 2-level HLM analyses tested outcomes as function of adherence to the treatment, controlling for all of the above child and family characteristics. Compared to children in the control group, children who received the expected dose of Educare scored significantly higher on PLS-4 Auditory Comprehension (*B* = 5.70, *se* = 2.33*, p* < 0.05, *d* = 0.25) and WJ-3 Applied Problems (*B* = 5.39, *se* = 1.94*, p* < 0.01, *d* = 0.28) and had significantly fewer BITSEA Problems (*B* = −3.30, *se* = 1.04, *p* < 0.01, *d* = −0.28).

**Table 6:** Per protocol analysis.

	PLS-4^a^ Eng AC		PLS-4 Span AC		EF total		WJ-3^b^ AP		BITSEA^c^ Problems		BITSEA^c^ Competence		Parent Positive		Parent Negative		Child Positive	
Fixed Effect	B	SE	B	SE	B	SE	B	SE	B	SE	B	SE	B	SE	B	SE	B	SE
Educare	5.70*	2.33	1.66	3.89	−0.03	0.02	5.39**	1.94	−3.30**	1.04	0.45	0.39	0.21	0.16	−0.06	0.14	0.09	0.14
Child age at entry	3.99	2.95	4.26	4.98	0.02	0.03	1.56	2.50	−1.23	1.30	−0.40	0.50	−0.32	0.21	0.44*	0.19	−0.22	0.19
Age at assessmt	−6.35	7.49	−22.82	13.32	0.03	0.08	−3.73	6.40	−0.22	3.35	−0.81	1.25	1.49**	0.55	−1.17*	0.51	0.73	0.57
Birth weight	1.65	1.11	−1.22	1.54	0.01	0.01	−0.05	0.91	0.26	0.47	−0.09	0.17	−0.08	0.07	0.07	0.06	−0.04	0.08
Depression	0.06	2.30	−0.10	3.84	0.02	0.02	−1.38	1.98	1.61	1.03	0.11	0.39	−0.05	0.16	0.06	0.14	−0.09	0.15
Mother educ	0.70	0.55	−0.81	0.73	0.01	0.01	0.78	0.46	−0.10	0.23	0.13	0.09	0.05	0.04	0.01	0.03	0.03	0.04
Mother hrs wrk	−0.06	0.07	0.15	0.12	0.00	0.00	−0.13*	0.06	0.00	0.03	0.00	0.01	0.00	0.00	0.00	0.00	0.00	0.00
Mother age	−0.32	0.18	0.18	0.27	0.00	0.00	−0.20	0.15	−0.16*	0.08	0.04	0.03	0.00	0.01	0.00	0.01	−0.01	0.01
Male	−5.19*	2.32	−0.67	3.81	0.01	0.02	−2.06	1.96	2.12*	1.01	−1.25**	0.39	−0.04	0.15	0.11	0.13	−0.18	0.14
DLL	−14.61**	5.47	–	–	−0.06	0.05	−13.00**	4.37	2.78	2.27	−1.26	0.92	−0.23	0.36	−0.13	0.31	−0.54	0.32
African American	3.14	5.92	−20.29	39.76	−0.02	0.05	−5.63	5.14	0.90	2.44	−0.14	0.89	−0.34	0.37	0.40	0.31	−0.45	0.37
Hispanic	6.23	3.85	9.82	5.18	0.04	0.04	−0.27	3.26	0.18	1.66	0.72	0.61	0.04	0.26	0.01	0.23	0.37	0.23
*Adjusted Means*																		
Control	90.07	2.41	142.19	46.66	0.39	0.02	84.57	2.03	12.01	1.28	18.39	0.30	4.89	0.12	1.72	0.11	4.95	0.15
Full protocol	95.76	2.38	143.85	3.89	0.36	0.02	89.95	2.07	8.71	1.28	18.84	0.39	5.10	0.16	1.66	0.14	5.04	0.14

a PLS-4 = Preschool Language Scale, 4th edition.

b WJ-3 = Woodcock-Johnson-3.

c BITSEA = Brief Infant Toddler Social Emotional Assessment.

* *p* < 0.05.

** *p* < 0.01.

#### 3.2.2 Moderators of treatment effects on child outcomes

The second research question was whether certain child characteristics moderate the effect of Educare on child outcomes; that is, whether Educare may be more or less effective for children of a specific race/ethnicity or children whose home language is not English. In this sample there is 91% overlap between Hispanic and DLL children. Therefore, DLL is included as a moderator and Hispanic is not. Table 7 presents results from the 2-level HLM controlling for child’s age at entry and at assessment, and mother’s hours worked, depression, education, and age. There were no significant interactions between Educare and race. However, there was a significant interaction between Educare and DLL status. On the PLS-AC English assessment, Educare impacts were larger for DLL children than for English-only children (DLL in Educare = 84.85, DLL not in Educare = 70.49, English-speaking in Educare = 94.52, English-speaking not in Educare = 90.02, *p* < 0.01, *d* = 0.73).

**Table 7:** Hierarchical linear model results: testing effect of moderators on outcomes.

	PLS-4^a^ English AC		PLS-4 Span AC		EF Total		WJ-3 AP^b^		BITSEA^c^ Problems		BITSEA Competence		Parent Positive		Parent Negative		Child Positive	
	B	SE	B	SE	B	SE	B	SE	B	SE	B	SE	B	SE	B	SE	B	SE
Intercept	90.02	2.33	117.19	49.28	0.39	0.02	84.68	2.36	12.11	1.04	18.37	0.31	4.88	0.12	1.72	0.12	4.93	0.14
Educare	4.49*	2.13	−11.18	43.30	−0.02	0.02	3.98*	1.79	−2.44**	0.94	0.36	0.35	0.14	0.14	0.02	0.15	0.09	0.15
Entry age	3.84	2.72	1.34	4.64	0.01	0.03	1.37	2.38	−1.94	1.17	−0.33	0.46	−0.20	0.20	0.19	0.17	−0.03	0.19
Age at assmnt	−10.82	6.67	−12.52	11.92	0.04	0.07	−5.46	5.39	3.22	3.11	−0.67	1.15	1.25*	0.55	−0.80	0.52	0.63	0.60
Birth weight	1.17	0.98	−0.21	1.55	0.00	0.01	0.04	0.79	−0.12	0.44	0.02	0.16	−0.06	0.06	0.04	0.06	0.01	0.07
Mat depression	1.69	2.13	2.10	3.74	0.02	0.02	−1.24	1.83	1.45	0.99	0.04	0.37	−0.05	0.16	0.09	0.14	−0.09	0.15
Mat education	0.78	0.49	−0.82	0.69	0.01	0.00	0.80	0.43	−0.08	0.22	0.10	0.08	0.07	0.03	−0.01	0.03	0.05	0.03
Mat work hours	−0.04	0.06	0.14	0.11	0.00	0.00	−0.11*	0.05	0.00	0.03	0.00	0.01	0.00	0.00	0.00	0.00	0.00	0.00
Mat age	−0.18	0.17	0.20	0.26	0.00	0.00	−0.14	0.14	−0.16*	0.08	0.03	0.03	0.01	0.01	0.00	0.01	−0.01	0.01
Male	−4.82*	2.12	1.16	3.66	0.01	0.02	−1.39	1.82	1.80	0.96	−1.03**	0.36	0.00	0.14	0.10	0.14	−0.16	0.14
DLL	−19.54***	5.17	–	–	−0.06	0.05	−13.19**	4.62	2.32	2.32	−1.13	0.86	−0.18	0.38	−0.06	0.38	−0.41	0.38
African American	2.01	5.26	−7.23	57.59	−0.01	0.04	−7.10	4.79	0.05	2.20	0.05	0.80	−0.27	0.36	0.68*	0.34	−0.38	0.38
Hispanic	5.42	3.58	8.62	4.94	0.03	0.03	−0.88	2.99	1.46	1.57	−0.03	0.56	−0.03	0.24	0.23	0.22	0.12	0.22
TX x DLL	9.87*	4.21	–	–	0.05	0.04	3.11	3.56	−3.27	1.90	0.91	0.70	0.21	0.29	−0.04	0.27	0.10	0.29
TX x AA	−8.13	4.24	−23.40	80.63	−0.03	0.04	−2.68	3.60	1.79	1.91	−0.34	0.71	−0.24	0.30	0.14	0.28	−0.14	0.29

a PLS-4 = Preschool Language Scale, 4th edition.

b WJ-3 = Woodcock-Johnson-3.

c BITSEA = Brief Infant Toddler Social Emotional Assessment.

* *p* < 0.05.

** *p* < 0.01.

*** *p <* 0.001.

#### 3.2.3 Treatment effects on changes in outcomes between ages 2 and 3

All of the age 3 outcomes except for math and EF were also administered at the age 2 visit and were analyzed to answer the third research question by examining treatment differences in change over time. Change scores were computed and are presented in Table 8. Table 9 shows the results of analyses using the same 2-level HLM described above. Results did not yield significant treatment/control group differences in change scores. However, examining the descriptive means suggests that Educare children declined over time on language skills relative to a normative sample, whereas the control group remained stable.

**Table 8:** Mean of change scores between age 2 and age 3 by treatment group.

	Control			Treatment		
	N	Mean	SD	N	Mean	SD
PLS-4^a^ Eng Aud Comp	87	0.06	17.85	108	−3.28	14.04
PLS-4 Span Aud Comp	32	−2.59	16.29	43	−7.51	18.17
BITSEA^b^ Problems	88	−2.53	6.30	110	−2.27	5.44
BITSEA Competence	88	0.55	2.58	110	0.24	2.23
Parent Positive	75	0.06	0.87	91	−0.05	0.93
Parent Negative	75	−0.21	0.95	91	−0.21	0.91
Child Positive	75	0.20	1.02	91	0.26	1.03

a PLS-4 = Preschool Language Scale, 4th edition.

b BITSEA = Brief Infant Toddler Social Emotional Assessment.

**Table 9:** Hierarchical linear model analyses: testing change scores from age 2 assessment to age 3.

	Change PLS-4^a^ English AC		Change PLS-4 Spanish AC		Change BITSEA^b^ Problems		Change BITSEA Competence		Change Parent Positive		Change Parent Negative		Change Child Positive	
	B	SE	B	SE	B	SE	B	SE	B	SE	B	SE	B	SE
Intercept	−0.26	2.09	−50.77	48.98	−2.32	0.76	0.42	0.30	0.18	0.15	−0.28	0.15	0.37	0.18
Educare	−3.42	2.28	−5.76	4.37	0.03	0.88	−0.26	0.35	−0.23	0.16	0.10	0.17	−0.13	0.19
Child age at entry	8.28**	2.79	−4.54	5.60	0.85	1.11	−1.06*	0.46	−0.36	0.22	0.31	0.20	−0.51*	0.21
Age at assessment	2.10	7.05	11.05	13.81	4.09	2.88	−0.33	1.14	1.04	0.63	−0.78	0.63	1.32*	0.67
Birth weight	2.10*	1.03	−1.08	1.75	−0.03	0.40	0.09	0.15	−0.09	0.07	−0.02	0.07	0.02	0.08
Mat depression	1.08	2.25	−1.15	4.30	−0.76	0.94	−0.23	0.36	−0.11	0.18	−0.05	0.16	0.01	0.18
Mat education	−0.13	0.52	−1.09	0.80	0.14	0.21	0.03	0.08	−0.01	0.04	0.00	0.04	0.04	0.04
Mat work hours	−0.09	0.07	0.11	0.13	0.02	0.03	0.00	0.01	0.00	0.01	−0.01	0.00	0.00	0.00
Mat age	0.03	0.17	−0.40	0.30	−0.05	0.07	0.04	0.03	0.00	0.01	0.00	0.01	−0.01	0.01
Male	−2.22	2.26	2.96	4.24	1.47	0.91	−0.24	0.36	0.05	0.16	0.15	0.16	−0.14	0.17
DLL	−10.58*	4.84	–	–	−0.58	1.94	0.60	0.77	0.03	0.38	0.41	0.39	−0.15	0.41
African American	−1.19	5.15	−21.30	46.36	−1.03	1.98	0.52	0.77	0.02	0.41	0.15	0.40	0.06	0.51
Hispanic	3.35	3.61	4.28	5.68	−0.18	1.36	−0.30	0.55	0.25	0.27	−0.01	0.25	0.29	0.27

a PLS-4 = Preschool Language Scale, 4th edition.

b BITSEA = Brief Infant Toddler Social Emotional Assessment.

* *p* < 0.05.

** *p* < 0.01.

#### 3.2.4 Type of care associations with child outcomes

Finally, two analyses addressed the fourth research question about whether children’s age 3 outcomes and age 2 to age 3 change scores differed depending on primary setting defined as Educare, other center-based care, or little or no center-based care. For these analyses, random assignment was ignored and children were assigned to a care group if they had attended Educare or other center-based programs 1,200 h or more as of age 3 (see Table 4 for primary groups). Table 10 shows the results of the 2-level HLM analyses that tested age 3 outcomes as a function of care group and controlled for site and all of the above child and family characteristics. Compared to children in the little/no care group, children in the Educare group scored significantly higher on PLS-4 Auditory Comprehension (*B* = 7.91, *se* = 2.59*, p* < 0.01, *d* = 0.39) and WJ-3 Applied Problems (*B* = 5.84, *se* = 2.19*, p* < 0.05, *d* = 0.41) and had significantly fewer BITSEA Problems (*B* = -3.92, *se* = 1.16, *p* < 0.01, *d* = 0.58). Compared to children in the little/no care group, children in the other center-based group scored significantly higher only on PLS-4 Auditory Comprehension (*B* = 8.24, *se* = 2.99*, p* < 0.05, *d* = 0.40). There were no significant differences on any outcomes between children in the Educare and other center groups.

**Table 10:** Hierarchical linear model results: testing age 3 effects by child care type.

	PLS-4^a^ English AC		PLS-4 Span AC		EF total		WJ-3^b^ AP		BITSEA^c^ Problems		BITSEA Competence		Parent Positive		Parent Negative		Child Positive	
Fixed Effect	B	SE	B	SE	B	SE	B	SE	B	SE	B	SE	B	SE	B	SE	B	SE
Intercept	86.79	2.58	107.57	40.74	0.37	0.02	83.72	2.09	13.27	1.12	18.20	0.35	4.72	0.14	1.85	0.13	4.87	0.15
Educare v little/no ctr care	7.91**	2.59	0.04	4.01	−0.01	0.02	5.84*	2.19	−3.92**	1.16	0.62	0.44	0.37	0.18	−0.19	0.16	0.17	0.17
Center care v little/no ctr care	8.24*	2.99	−5.77	5.91	0.02	0.03	1.79	2.53	−2.16	1.34	0.10	0.51	0.20	0.21	−0.07	0.18	0.02	0.19
Educare v. other center care	−.32	2.66	5.81	5.71	−.03	.03	4.04	2.26	−1.67	1.1	0.52	0.45	0.17	0.18	−0.11	0.17	0.15	0.18
Age	−7.17	6.68	−11.24	11.83	0.04	0.06	−3.77	5.53	1.35	2.98	−0.23	1.11	1.37*	0.57	−0.86	0.51	0.67	0.59
Child age at entry	4.93	2.70	0.86	4.56	0.02	0.03	1.33	2.29	−2.34*	1.21	−0.26	0.46	−0.20	0.20	0.17	0.17	−0.03	0.19
Birth weight	1.20	0.99	−0.46	1.52	0.00	0.01	0.06	0.82	−0.10	0.44	0.01	0.16	−0.07	0.07	0.05	0.06	0.01	0.07
Mother depression	1.08	2.16	2.43	3.72	0.02	0.02	−1.22	1.83	1.95*	0.97	−0.13	0.37	−0.08	0.15	0.10	0.14	−0.11	0.14
Mother educ	0.80	0.50	−0.75	0.68	0.01	0.00	0.87*	0.42	−0.08	0.22	0.09	0.08	0.07*	0.03	−0.01	0.03	0.05	0.03
Mother work hr	−0.04	0.06	0.14	0.11	0.00	0.00	−0.12*	0.05	0.00	0.03	0.00	0.01	0.00	0.00	0.00	0.00	0.00	0.00
Mother age	−0.24	0.17	0.19	0.25	0.00	0.00	−0.17	0.14	−0.14	0.07	0.03	0.03	0.01	0.01	0.00	0.01	−0.01	0.01
Male	−5.56*	2.16	1.19	3.63	0.01	0.02	−2.10	1.83	2.08*	0.97	−1.08**	0.37	−0.05	0.15	0.13	0.13	−0.19	0.14
DLL	−11.88*	4.80	–	–	−0.04	0.04	−10.06*	4.03	0.48	2.12	−0.78	0.79	−0.03	0.36	−0.15	0.33	−0.28	0.36
African American	3.61	5.10	−20.39	31.34	−0.02	0.04	−3.73	4.44	0.41	2.16	−0.21	0.75	−0.27	0.35	0.65*	0.31	−0.31	0.36
Hispanic	4.82	3.41	8.99	4.81	0.03	0.03	−1.64	2.87	1.54	1.53	0.03	0.57	−0.08	0.24	0.27	0.21	0.09	0.23
*Adjusted Means*																		
Educare	94.72	2.21	88.68	2.94	0.36	0.01	89.54	1.76	9.27	0.94	18.83	0.26	5.10	0.11	1.66	0.09	5.04	0.12
Other center care	95.05	2.70	82.87	5.38	0.39	0.02	85.50	2.20	11.03	1.17	18.31	0.37	4.93	0.15	1.77	0.14	4.90	0.16
Little/no care	86.81	2.58	88.63	3.48	0.37	0.02	83.70	2.08	13.19	1.12	18.21	0.35	4.73	0.14	1.84	0.13	4.87	0.15

a PLS-4 = Preschool Language Scale, 4th edition.

b WJ-3 = Woodcock-Johnson-3.

c BITSEA = Brief Infant Toddler Social Emotional Assessment.

* *p* < 0.05.

** *p* < 0.01.

The same 2-level HLM was also conducted on the change scores of the Educare, other center-based care, and little/no care group between ages 2 and 3. While the model was not statistically significant for any of the outcomes, the age 2 to age 3 adjusted PLS-4 scores decreased more than 3 points for both the Educare and the little/no care groups and increased 2.5 points for the other center-based care group. This convergence seems to account for the lack of significant differences in age 3 scores between Educare and other center-based care.

## 4 Discussion

This study extended a previous randomized study of a high-quality early education program for infants and toddlers by reporting outcomes for children at age 3. The study provides further evidence of the benefits of investing early in children’s lives, although the results at age 3 were more modest than the results at age 2. The effect sizes were, however, about twice the size of those in the Early Head Start randomized study at age 3 (Love et al., 2005). In ITT analyses, Educare children scored higher than controls on English language and math scores and had fewer parent-reported behavior problems, with stronger English language results for DLL children. Effect sizes for significant findings were in the modest range (*d* = 0.24–0.35). Results of the per protocol analysis were very similar, adding confidence to the findings. Change scores from age 2 to age 3 suggested that the groups may be converging.

In analyses examining primary child care setting types, regardless of group assignment, children who attended Educare or other center-based programs scored better than children who had little or no formal child care on measures of English language and math skills and had fewer parent-reported behavior problems. However, children who attended other center-based child care programs also scored higher on English language skills than the little/no care group. Effect sizes between the little/no care group and both Educare and other center-based care were in the modest to medium range (*d* = 0.40–0.58). There were no significant differences between children in the Educare and other center-based care groups on any of the measures.

### 4.1 Sustained and new effects

The present study extends the previous findings at age 2 (Yazejian et al., 2017) by showing that Educare continues to affect children’s language and social-emotional skills at age 3, and now can be shown to impact children’s early math skills. These findings support the notion that high-quality ECE can promote school readiness skills for children from low-income families (Burchinal et al., 2000, Dearing et al., 2009, McCartney et al., 2010, Votruba-Drzal et al., 2013).

Effects of Educare on English language skills at age 3 were greater for Spanish-speaking DLLs than for their English-speaking peers, an effect not found at age 2. There were no significant differences in effects for African-American children. While previous research has shown that DLL preschoolers may especially benefit from center-based care (Gormley, 2008; Magnuson, Meyers, Ruhm, & Waldfogel, 2004; Morris et al., 2018; Padilla & Ryan, 2018; Puma et al., 2010), there have been few studies examining the experiences and outcomes of DLL infants and toddlers in ECE settings (Buysse et al., 2014). We know that Spanish-speaking DLLs from low-income families start kindergarten behind their peers in reading skills (Reardon & Galindo, 2009) and that their English language skills at school entry predict achievement through middle grades (Halle, Hair, Wandner, McNamara, & Chien, 2012; Han, 2012; Kieffer, 2012), but their trajectory of language development from birth to age 4 when enrolled in ECE is less well documented. Most Educare DLLs were in a classroom with a bilingual adult, but most daily activities in the schools in this study were in English. This English exposure and practice likely facilitated their language acquisition with no significant decreases in Spanish language skills from age 2 to age 3. Given the large number of control children enrolled in other center-based care, the same may have been true for them (Espinosa, 2013).

In the social-emotional domain, the present study found that children in Educare were rated by parents as having fewer problem behaviors than children in the control group, an effect size 3 times that of the EHS random study (d = −0.35 v.−.11; Love et al., 2005). Because we have only parent-reported measures, we do not know whether this difference reflects variations in parent perceptions or actual child functioning. Previous findings related to the link between high-quality ECE and social-emotional outcomes have been mixed. Recent research suggests a bidirectional association between social-emotional skills, such as self-regulation, and language and literacy outcomes (Skibbe, Montroy, Bowles, & Morrison, 2018), highlighting the need to understand associations among ECE, language, and social-emotional skills. Additional research, with direct measures of children’s functioning, is needed.

Children in the treatment group also had better early math skills (*d* = 0.28). Four of the 5 Educare schools in this study have adopted a specific preschool math curriculum in addition to their broad, general curriculum. However, the research-based curricula for young children target 3- to -5-year olds (Clements & Sarama, 2007), not infants and toddlers, and we have no additional data about how much time is spent on math or how math is taught in Educare. The average preschool teacher spends little time on math (Clements and Sarama, 2007, Early et al., 2010), but the Educare group’s math results indicate that they may be getting more than most.

### 4.2 Null effects

The lack of an effect on EF is not unique to the current study. A review of studies with school-age children failed to find an effect on EF for broad interventions (Jacob & Parkinson, 2015), although some studies suggest that high-quality pre-K programs can improve EF (Burchinal, Vernon-Feagans, Vitiello, & Greenberg, 2014; Weiland & Yoshikawa, 2013). We know of no studies that have examined impact as young as age 3. Most EF tasks are difficult for preschoolers, especially 3-year-olds. Compared to 3-year-olds from a mixed SES sample (Kuhn, Camerota, Willoughby, & Blair, 2020, under review), children in this study had relatively low scores. Some children were unable to meet basal requirements on any of the tasks and only a few met them on all tasks.

The significant differences at age 2 between treatment and control groups in sensitive parent-child interactions (Yazejian et al., 2017) were not found at age 3. Parent engagement is a key component of Educare with its goal to encourage positive parent-child relationships; however, we do not have detailed information about the nature of parent engagement activities and how those activities may change as children age. Perhaps programs use more effective strategies for parents of infants and toddlers, or perhaps parents of toddlers are more receptive to interactions with program staff than they are when their toddler has become a more mature preschooler.

### 4.3 Possible convergence

The magnitude of language differences previously reported for this sample when children averaged age 2 (*d =* 0.58; Yazejian et al., 2017) was larger than the age 3 results reported here (*d* = 0.24). Analyses did not reveal significant differences between treatment and controls in change scores from ages 2–3, but the reduced magnitude of effect suggests that the language skills of the groups are converging. Both groups had equivalent scores at entry (mean age = 9 months), although infant language assessments may be unreliable. From baseline to age 2, the control group had a decline in language standard scores as is unfortunately typical for low-income children (Halle et al., 2009), while the treatment group made small gains (Yazejian et al., 2017)–suggesting that Educare children were making gains comparable to the norming sample, which is more advantaged on average. However, from ages 2–3, average language scores of the treatment group declined relative to the norming sample while the control group remained constant. During this time, use of center-based care by control families increased. At age 2, 47% of control children had attended or were attending center-based care; at age 3, this had increased to 64%, including some children (14%) in EHS/HS or pre-K programs. The ECE experiences of the control group may explain the reduced magnitude of effect between the groups on language outcomes (Morris et al., 2018). The counterfactual in this study differed greatly from that of decades-earlier randomized studies of infant-toddler interventions, in which the control group was much less likely to experience center-based care by age 3.

### 4.4 Child care experiences

When we ignored random assignment and compared children who attended Educare for at least a year with children who attended other center-based programs for at least a year and with children who had little or no care, the findings were surprising. As more control children entered center-based care between ages 2 and 3, we expected that their language and behavior scores might increase, but the fact that there were no significant differences in scores between children in Educare and other center-based care at age 3 was not expected. Nineteen of the 49 children in the center-based care group (39%) had Early Head Start or some type of public pre-k program experience and many, if not most, of the other center-based children were in programs that were regulated at some level. However, we do not know the quality of those learning environments. Educare school leaders are noted for their community advocacy for programs and policies to help better serve all vulnerable children and families, and the philanthropies that support Educare in these cities also support other ECE programs. Perhaps these efforts have helped the overall standard of care in these communities to improve.

Our finding that children primarily in informal care settings or cared for at home by parents had lower scores on measures of language than children attending either Educare or other center-based care has been found in two re-analyses of data from the HS Impact Study (Kline and Walters, 2016, Morris et al., 2018). When the counterfactual is parent or informal care, the impact of HS is significant. It would also seem that Educare’s advantage appears mostly in comparison to children cared for by parents or in informal care. The high-quality intervention may not confer significant benefits to children beyond those obtained in the typical center-based care available in the study communities, which have widely available public pre-k programs and philanthropic benefactors whose support for quality ECE extends beyond Educare.

### 4.5 Limitations

Several limitations of this study should be noted. First, attrition was an issue, especially for the control group. Overall, 30 of 121 control and 7 of 118 treatment children withdrew or could not be located at age 3. Two baseline background characteristics—birthweight and Hispanic ethnicity—differed between treatment and control groups at age 3. However, children no longer in the study had lower baseline language scores, and their mothers were more likely to have screened positive for depression, compared those who remained in the study at age 3. This may have decreased the potential magnitude of effects as well as generalizability. Differential attrition is a common problem in longitudinal studies of children from low-income families (e.g., Carlson, 2009). In addition, the reduced sample size at age 3 reduced the power to detect small effects.

Second, while cross-overs were not a large problem in this study, four children assigned to Educare did not receive the intervention at all, and one child assigned to the control group entered Educare at age 3. The study was originally designed as a birth-to-age-3 study, with families’ understanding that, if assigned to the control condition, their child could not enter Educare before age 3. When funding became available to follow children to age 5, the original agreements with programs and families were honored.

Due to attrition and some children not receiving the full dose of Educare as described above, both the ITT and PP analyses were under powered to detect small to moderate effect sizes. Attrition was greater for children in the control group than children enrolled in Educare and this limitation may have led to imprecise estimates of treatment effects. Likewise, this study was also underpowered for subgroup analyses. In particular, the DLL and African-American subgroup analyses involved smaller sample sizes and were less powered to detect small and moderate effect sizes, but these analyses were included given previous findings and the importance of understanding differential treatment effects. Having a large enough sample to detect small effect sizes is particularly relevant in the area of ECE given that historically significant effects have been in the small to moderate range.

Third, more detailed information about the experiences of children in the control group, as well as details on program implementation in both treatment and control conditions, were not gathered because of budget constraints. While we were able to categorize children into types of child care attended, we did not have observations to know about the quality of their experiences in those settings. We also know relatively little about the parent engagement components of the settings. More measures about the extent to which programs, both Educare and other center-based settings, implement components of Educare would assist in identifying the potential mechanisms of the Educare intervention and other high-quality ECE programs.

Fourth, Educare does not provide transportation and therefore is likely not serving children and families who may be most disadvantaged and isolated from services. In locations without regular, accessible public transportation, having a car might be required for maintaining both work/school requirements of subsidy receipt and attendance requirements for Early Head Start/Head Start. The results of this study therefore may not generalize to children and families most in need of educational and family support services. However, access to reliable transportation, if needed, was assessed as part of determining program eligibility, before random assignment, and therefore did not contribute systematically to any treatment-control differences.

Fifth, although random assignment was employed to ensure that any differences found between groups could be attributed to the treatment, descriptive analyses revealed that children in the control group had significantly lower birthweights, on average, than children in the treatment group at baseline. While we included birthweight as a covariate in all analyses to control for the difference, this nevertheless should be considered a potential limitation of the study.

Finally, there are limitations related to measurement. Some researchers and practitioners question the ability of the PLS-5 to provide an accurate measure of language skills and also think it contains cultural and linguistic biases that make it especially inappropriate for diverse populations (Crowley, 2013). This criticism would not seem to have biased results in favor of one group or another in our study, but it may be that the measure is not sensitive to intervention effects. In addition, only parent-report measures of social-emotional skills were included. The finding of an effect on children’s behaviors may reflect the effect of the Educare intervention on children, an effect of Educare programming on parent perceptions of behavior, or both. The next phase of the study will include teacher report of social skills when children are 5 years old.

### 4.6 Conclusions, implications, and next steps

The results of this study showed thsat Educare continues to have a positive effect on children’s development at age 3. However, because the treatment and control groups appear to be converging over time, and the treatment effect seems to be driven by differences between children in Educare compared to children with little/no formal care, questions remain about the nature of children’s experiences in various settings. Future studies should fully document classroom and family engagement aspects of early childhood programming to understand mechanisms of associations between children’s experiences and outcomes.

Current study funding will allow follow-up of children through age 5 with cognitive, language, social-emotional, and pre-academic measures. This study is one of several ongoing efforts within the research-practice partnership that is the Educare Learning Network to answer questions of interest to Educare practitioners and the wider ECE field. Along with an ongoing implementation study that gathers data on Educare children and several studies that follow Educare graduates into school, the age 5 follow-up of the current experimental study sample will help determine whether children who attend Educare have language and social-emotional advantages that might predict longer term benefits in school readiness.

## Funding

We gratefully acknowledge the funding support for this work provided by the Buffett Early Childhood Fund, the Brady Education Foundation, the George Kaiser Family Foundation, the Bill & Melinda Gates Foundation (#51976), and an anonymous foundation. The content of this publication does not necessarily reflect the views or policies of these foundations. We also thank Diane Webster, John Cashwell, and David Gardner for their meticulous assistance with the data for this paper. We thank Melinda Berry, Linda Henson, Kathy Jones, Ashley Midthun, Elizabeth Miranda, Megan Shepherd, Amanda Treptow, Deb Winkelmann, and Lauren Worley for their diligence in recruiting families, gathering data, and tracking families over time through ongoing communication. Finally, we thank the Educare programs and staff, families, and children who participated.

## CRediT authorship contribution statement

**Noreen Yazejian:** Conceptualization, Methodology, Writing - original draft, Supervision, Funding acquisition. **Donna M. Bryant:** Conceptualization, Methodology, Writing - original draft, Supervision, Funding acquisition. **Laura J. Kuhn:** Formal analysis, Writing - original draft. **Margaret Burchinal:** . **Diane Horm:** Conceptualization, Methodology, Writing - review & editing, Supervision, Funding acquisition. **Sydney Hans:** Conceptualization, Methodology, Writing - review & editing, Supervision, Funding acquisition. **Nancy File:** Conceptualization, Methodology, Writing - review & editing, Supervision, Funding acquisition. **Barbara Jackson:** Writing - review & editing, Supervision.
